# Integrating sexual and reproductive health into health system strengthening in humanitarian settings: a planning workshop toolkit to transition from minimum to comprehensive services in the Democratic Republic of Congo, Bangladesh, and Yemen

**DOI:** 10.1186/s13031-020-00326-5

**Published:** 2020-11-25

**Authors:** Nguyen Toan Tran, Alison Greer, Brigitte Kini, Hassan Abdi, Kariman Rajeh, Hilde Cortier, Mohira Boboeva

**Affiliations:** 1 Australian Centre for Public and Population Health Research, Faculty of Health, University of Technology Sydney, PO Box 123, Sydney, NSW 2007 Australia; 2 Faculty of Medicine, University of Geneva, Rue Michel-Servet 1, 1206 Genève, Switzerland; 3 Training Partnership Initiative of the Inter-Agency Working Group on Reproductive Health in Crises, Women’s Refugee Commission, 15 West 37th Street, New York, NY 10018 USA; 4 World Health Organization Country Office in the Democratic Republic of Congo, Avenue des Cliniques 42, BP 1899 Kinshasa I, Democratic Republic of Congo; 5 Consultant, PO Box 617, Garissa, 70100 Kenya; 6 World Health Organization, PO Box 543, Sana’a, Yemen; 7 Consultant, Tuinbouwlaan 30, 1700 Dilbeek, Belgium; 8 World Health Organization, Global Health Cluster, Avenue Appia 20, 1211, 27 Geneva, Switzerland

**Keywords:** Health system strengthening, Minimum initial service package (MISP) for sexual and reproductive health, Comprehensive services, Participatory, Planning toolkit

## Abstract

**Background:**

Planning to transition from the Minimum Initial Service Package for Sexual and Reproductive Health (SRH) toward comprehensive SRH services has been a challenge in humanitarian settings. To bridge this gap, a workshop toolkit for SRH coordinators was designed to support effective planning. This article aims to describe the toolkit design, piloting, and final product.

**Methods:**

Anchored in the Health System Building Blocks Framework of the World Health Organization, the design entailed two complementary and participatory strategies. First, a collaborative design phase with iterative feedback loops involved global partners with extensive operational experience in the initial toolkit conception. The second phase engaged stakeholders from three major humanitarian crises to participate in pilot workshops to contextualize, evaluate, validate, and improve the toolkit using qualitative interviews and end-of-workshop evaluations. The aim of this two-phase design process was to finalize a planning toolkit that can be utilized in and adapted to diverse humanitarian contexts, and efficiently and effectively meet its objectives. Pilots occurred in the Democratic Republic of Congo for the Kasai region crisis, Bangladesh for the Rohingya humanitarian response in Cox’s Bazar, and Yemen for selected Governorates.

**Results:**

Results suggest that the toolkit enabled facilitators to foster a systematic, participatory, interactive, and inclusive planning process among participants over a two-day workshop. The approach was reportedly effective and time-efficient in producing a joint work plan. The main planning priorities cutting across settings included improving comprehensive SRH services in general, healthcare workforce strengthening, such as midwifery capacity development, increasing community mobilization and engagement, focusing on adolescent SRH, and enhancing maternal and newborn health services in terms of quality, coverage, and referral pathways. Recommendations for improvement included a dedicated and adequately anticipated pre-workshop preparation to gather relevant data, encouraging participants to undertake preliminary study to equalize knowledge to partake fully in the workshop, and enlisting participants from marginalized and underserved populations.

**Conclusion:**

Collaborative design and piloting efforts resulted in a workshop toolkit that could support a systematic and efficient identification of priority activities and services related to comprehensive SRH. Such priorities could help meet the SRH needs of communities emerging from acute humanitarian situations while strengthening the overall health system.

## Background

The Minimum Initial Service Package (MISP) for Sexual and Reproductive Health (SRH) is a priority set of life-saving interventions to be implemented at the onset of every humanitarian crisis [1]. The first objective of this international standard in humanitarian response is to ensure that the health sector or cluster identifies an organization to lead the implementation of the MISP [2]. The second objective is to prevent sexual violence and respond to the needs of survivors. The third objective is to prevent the transmission of and reduce morbidity and mortality due to HIV and other sexually transmitted infections. The fourth objective is to prevent excess maternal and newborn morbidity and mortality. The fifth objective is to prevent unintended pregnancies, and the last and sixth objective is to *plan* for comprehensive SRH services that are integrated into primary healthcare. The implementation of comprehensive SRH services follows the planning process and falls outside of the MISP. Here, *integration –* the terminology used in the MISP guidance – refers to the inclusion of comprehensive SRH services into a package of primary healthcare services rather than integrating services, such as integrating postpartum family planning with child immunization programs. The MISP also notes the importance of ensuring that safe abortion care is available to the full extent of the law, as another priority.

Planning for comprehensive SRH should occur as soon as possible and in close coordination with the health sector or cluster partners at the national or subnational level according to the humanitarian and country contexts. Strengthening the six health system building blocks as defined by the World Health Organization (WHO) offers a useful planning framework to inform the inclusion of comprehensive SRH services into a primary healthcare package and reinforcement of other comprehensive SRH services at the higher levels of care [3]. These blocks encompass service delivery, the health workforce, health information system, medical commodities, financing, and governance and leadership. Therefore, planning should involve the various components of the health system as well as other sectors and ministries, such as the education and finance ministries.

Since the conception of the MISP in the mid-1990s, progress has been made in scaling up SRH services in crisis-affected settings [4]. For example, the global awareness of the MISP increased, and implementing MISP-related clinical services made headway, such as HIV prevention and gender-based violence prevention and care for survivors [5]. However, there are still critical gaps, including the challenge in implementing the MISP fully and systematically, the limited transition from the MISP to comprehensive SRH services and service integration into primary healthcare as the situation stabilizes, and insufficient attention to adolescent SRH needs [6].

**Fig. 1:**
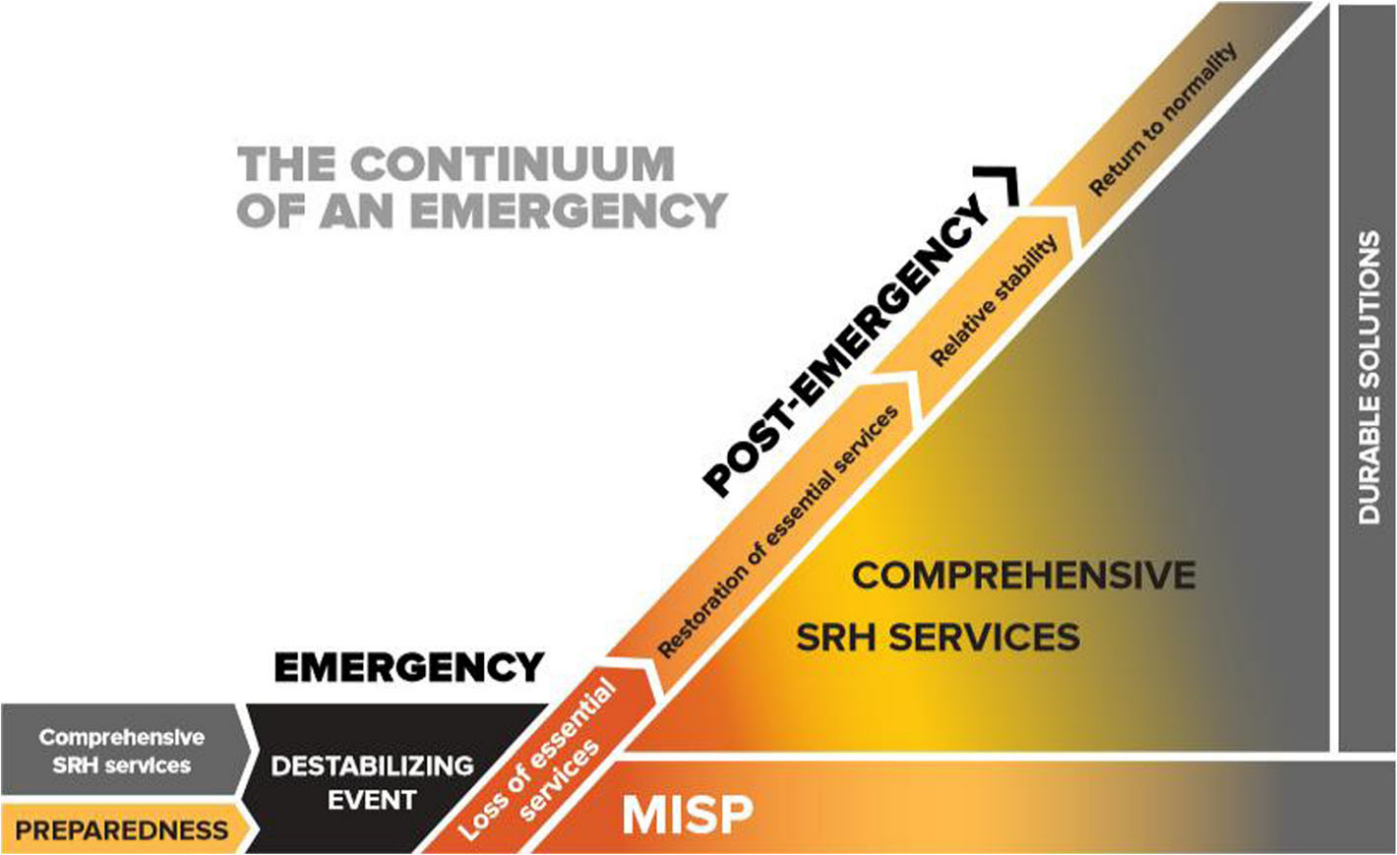
The Minimum Initial Service Package (MISP) for Sexual and Reproductive Health (SRH) and comprehensive SRH services within the continuum of an emergency. Source: Inter-agency Field Manual on Reproductive Health in Humanitarian Settings 2018

Experience has shown that Objective 6 of the MISP remains challenging to implement [7]. It requires long-term vision, leadership, effective coordination skills, and a sound understanding of the local situation and opportunities related to health system strengthening. As highlighted in Objective 6, *the clinical services of the MISP should be sustained, improved in quality, and expanded upon with other comprehensive SRH services and programming throughout protracted crises and the recovery and reconstruction phases* [8]. Comprehensive SRH builds upon the MISP as described in Fig. 1. Its planning and implementation must occur as soon as possible as assessed by field actors, allowed by the situation, and, importantly, without a prescribed timing as of when the process should start after the onset of the crisis.

Priorities for achieving comprehensive SRH include improving the quality and coverage of MISP services as well as including SRH services that fall outside of the MISP. According to the 2018 Guttmacher-Lancet Commission, comprehensive SRH services are *essential sexual and reproductive health services that must meet public health and human rights standards, including the ‘Availability, Accessibility, Acceptability, and Quality’ framework of the right to health* [9]*.* Examples of such services encompass, among others, accurate information and counseling on sexual and reproductive health, including evidence-based, comprehensive sexuality education; prevention, detection, and treatment of reproductive cancers; and information, counseling, and care related to sexual function and satisfaction.

To fully achieve Objective 6 of the MISP and support national and local stakeholders and, where needed, international actors, in the initial planning for the delivery of comprehensive SRH services, there must be consideration of several critical aspects. Such aspects, often challenging to implement in humanitarian, insecure, and under-resourced settings, include communication among decision-makers (including national governments) and implementing partners, adequate financing, effective coordination, supply chain management, human resources management, monitoring and evaluation, and a system of information sharing, feedback, and meaningful participation of and accountability to the affected community. As with the MISP, comprehensive SRH services must be of good quality and accessible for all crisis-affected populations, including adolescents, unmarried as well as married women and men, persons living with disabilities, and lesbian, gay, bisexual, queer, questioning, intersex, and asexual people [10, 11].

To catalyze the planning process and bridge the nexus between the acute humanitarian response and post-acute development phase, the Training Partnership Initiative of the Inter-Agency Working Group (IAWG) on Reproductive Health in Crises, with the WHO Global Health Cluster, started designing a workshop toolkit in 2017. The toolkit objective is to support SRH coordinators and stakeholders in their efforts to plan for comprehensive SRH, with the understanding that Objective 6 of the MISP is not about implementing comprehensive SRH services but its programmatic *planning*. This article aims to describe the toolkit design, the piloting, and the final product.

## Methods

### Design framework and assumptions

With a view to strengthening health systems in humanitarian contexts and facilitate the inclusion of comprehensive SRH services into a primary healthcare package and higher levels of care, we grounded the toolkit into the WHO Health System Building Blocks Framework [12]. The blocks encompass governance and leadership, healthcare workforce, financing, products and supplies, health information system, and services.

Due to the complexity of humanitarian coordination combined with the socio-economic, cultural, political, and religious challenges and opportunities surrounding SRH, our design assumed that following participatory action research principles with two complementary phases would be essential to meet the toolkit objective and gain insights into the workshop implementation process [13]. First, a design phase with iterative feedback loops aimed to involve members of the IAWG Training Partnership Initiative, the wider IAWG coalition, and the WHO Global Health Cluster in the conception and finalization of the toolkit. Second, a bottom-up approach aimed to engage stakeholders from diverse crisis-affected, geographic, linguistic, and cultural contexts to participate in pilot workshops to improve successive toolkit drafts and enrich the document with best practice recommendations.

Authors also applied a participatory approach to the planning process methodology within the toolkit, assuming that it would be more effective than a top-down approach in fostering mutual understanding and coordination among planners. This is achieved by gathering relevant stakeholders, stimulating collective planning, giving ample space to exchange ideas and share positive and negative experiences and practices, and identifying gaps and opportunities together [14]. This momentum would then bring participants to reach a consensus on priority comprehensive SRH activities through individual reflection and group deliberations [15]. The implementation of the resulting plan would be maximized as stakeholders would own and mobilize resources for it [16].

### Design phase

There was no existing tool focused on the planning from MISP to comprehensive SRH programming based on the IAWG’s collective knowledge of the MISP. This was confirmed in January 2017 when we reviewed the literature on Google Scholar and Google Search using word strings or equivalents combining *reproductive health*, *MISP, humanitarian, transition,* and *comprehensive services.* We proceeded with the design process, which first engaged members of the IAWG who had extensive operational experience, including in facilitating the SRH response coordination as well as recovery planning in complex emergencies, and who struggled over the years with conveying what is lifesaving and comprehensive SRH [17]. Drawing from their MISP implementation practice and capacity development experience related to the coordination of the MISP and MISP-related clinical services, authors outlined the contents of the toolkit before extending successive drafts to other IAWG members [18]. This approach allowed an iterative refinement of the different steps proposed in the toolkit, which was purposefully constructed to be flexible, rather than prescriptive, to allow for adaptations to a variety of contexts.

#### Toolkit

The workshop toolkit, available at www.iawg.net, aims to guide SRH coordinators (or health coordinators and managers) along with national counterparts in facilitating a workshop to catalyze participatory planning among local stakeholders and partners through the development of a collective work plan for comprehensive SRH programming. The workshop could occur at the national or subnational level, depending on the context. Representatives from the following institutions should be considered for the workshop: not only the Ministry of Health but also the Ministries of Finance (for health services financing), Education (for health staff training and certification), and Home Affairs (for forcibly displaced populations); local, national and international humanitarian and development agencies and non-governmental organizations working on SRH-related services; communities of concern (e.g., adolescents, persons with disabilities, sex workers); women’s organizations; and professional associations (e.g., midwifery, nursing), among others. Table 1 gives an overview of the successive steps and related objectives to be undertaken before, during, and after the workshop. The toolkit describes essential information about the duration, overall approach, materials to prepare, and facilitation sequence for each step. The toolkit implementation process should span over at least 6 weeks of preparation, followed by a two-day to a three-day workshop and immediate post-workshop activities.

**Table 1:** Steps, objectives, and methodology of the participatory planning workshop toolkit

Steps	Objectives	Methodology
Pre-workshop preparation	To prepare background documents to inform the discussions during the workshop (mapping of the implementation of the MISP and comprehensive SRH services, relevant policies, barriers, opportunities, and key stakeholders)	Collection of background SRH data and mapping of key stakeholders using pre-defined templates
Introductions and expectations	To break the ice among participants and agree on the objectives of the workshop	Interactive plenary discussion
Step 1 - Setting a common understanding	To set the scene for the workshop with an overview of the essential information that participants need to be aware of in order to plan for comprehensive SRH effectively	Interactive PowerPoint presentations
Step 2 - Mapping needs and opportunities related to comprehensive SRH	To reflect upon, discuss, and map current needs and opportunities in relation to comprehensive SRH programming	Personal reflection using post-its and work in small and large groups using a common and pre-established wall chart to capture the personal reflections according to needs, opportunities and the six health system building blocks
Step 3 - Setting planning priorities for comprehensive SRH	To agree on a set of planning priorities related to comprehensive SRH	Using a pre-established wall chart, prioritization exercise according to the degree of urgency and required resources; sticky dots to cast individual votes; reflection and debate in small and large groups
Step 4 - Teamwork on agreed planning priorities for comprehensive SRH	To produce a detailed and practical work plan to implement the top three SRH priorities	Teamwork using a pre-established matrix
Step 5 - Reporting back and finding synergies	To establish a consolidated national (or provincial or sub-provincial, depending on the context) work plan to implement priority interventions related to comprehensive SRH	Group discussion
Post-workshop follow-up	To ensure that plans are followed through and challenges are addressed	As needed: follow-up meetings, emails, etc.

### Toolkit contextualization and validation

Intending to inform the design of the toolkit further and ensure its relevance to the diverse humanitarian contexts, we sought the participation of other IAWG colleagues and country teams operating in humanitarian contexts to pilot the toolkit and answer the following questions:

- Was the workshop toolkit fit for purpose, i.e., did it catalyze participatory planning to transition from the MISP to comprehensive SRH programming?
- What were the key lessons learned to improve the workshop toolkit?

To answer these questions, we conducted a review and synthesis of the workshop reports and evaluations, focusing on the structure and contents of the toolkit implementation. The analysis applied the following guiding and interlinked lenses:

- Effectiveness: to what extent was the pilot of the toolkit successful in producing the desired result, which is a plan for comprehensive SRH programming? Here, effectiveness does not refer to effectiveness evaluation regarding the work plan implementation and whether it has resulted in improved health outcomes.
- Efficiency: to what extent was the toolkit methodology considerate of time, effort, and human resources, which are known to be scarce in humanitarian contexts?
- Participation: to what extent was the workshop inclusive of key stakeholders?

Learnings from the process of implementing the toolkit were also captured and integrated into the toolkit. For example, the criteria used to prioritize SRH services may differ depending on the setting.

#### Contexts

The pilots occurred in August 2018 in the Democratic Republic (DR) of Congo, focusing on the Kasai region crisis, which affected local and internally-displaced populations; in November 2018 in Bangladesh, focusing on the humanitarian response for Rohingya refugees in Cox’s Bazar; and in February and March 2019 in Yemen focusing on local and internally-displaced populations in Ibb, Dhamar, and Aden Governorates. All three settings were under the system-wide level-3 (L3) emergency status, which concerns extreme crises beyond the capacity of local players and governments to respond. Note that the toolkit was not specifically designed for L3 settings but any humanitarian situation undertaking the planning for comprehensive SRH services.

The Kasai Provinces in DR Congo experienced violent tribal conflicts, which started in August 2016 and forcibly displaced internally an estimated 1.4 million people. The system-wide L3 status was activated in October 2017 and deactivated in April 2018 [19]. In August 2017, Rohingya Muslims from the northern areas of Rakhine State in Myanmar fled en masse to Bangladesh in response to violence committed by the Myanmar Army and the State. The system-wide L3 activation occurred in September 2017. In November 2018, a month before the planning workshop, there were over 900,000 refugees in Cox’s Bazar [20]. Fighting in Yemen, already one of the lowest income countries in the Middle East, intensified in late March 2015 and severely compounded humanitarian needs from long years of protracted poverty and insecurity. In July 2015, an L3 emergency was declared for the country. In January 2019, a month before the planning workshop, there were 24.1 million Yemenis in need of humanitarian assistance, among whom 3.3 million were internally displaced [21].

#### Participants

In each country, at the national and subnational levels, health authorities, in coordination with the WHO and United Nations Population Fund (UNFPA) country and field offices, identified the workshop participants. A majority came from the provinces or governorates and local, national, and international non-governmental organizations as well the midwifery association in Yemen. For the Kasai region workshop, there were 27 participants with 14 different affiliations, and in Cox’s Bazar, 39 participants and 20 affiliations. Political and logistical considerations dictated the organization of two separate workshops in Yemen. The first workshop took place in Sana’a (covering Ibb and Dhamar Governorates) with 36 participants (11 affiliations) and the second in Aden with 37 participants (13 affiliations). Although the toolkit emphasizes the need to include representatives from the community and marginalized groups as well from other Ministries, none of the workshops managed to do so due to time and other logistics constraints.

#### Collecting insights

At the beginning of each workshop, the participants received information about the participatory methodology within the toolkit and its design, the evaluation of the pilot workshops, and the publication and dissemination of subsequent results and best practices. In addition to end-of-workshop written evaluations that took place across settings, sufficient time allowed facilitators in Kinshasa and Sana’a to invite participants to a focus group discussion. The qualitative discussion guide followed the successive workshop steps and asked attendees to share related experiences, ideas, comments, suggestions, and recommendations to help improve the toolkit contents and methodology [22]. The workshop evaluation was part of planned program monitoring, which was not designed to develop and contribute to generalizable knowledge and therefore did not constitute research and require ethical approval [23]. Participants were informed that all their feedback would be anonymized, and its management and analysis handled confidentially. They were free to participate in the evaluation and were informed that they could withdraw at any time without consequences on their participation in the workshop. The evaluation was deemed to pose no risk to participants who had the opportunity to ask questions and receive clarifying comments before providing their written informed consent to being photographed, filmed, or audio recorded. There was no refusal across settings.

#### Synthesizing feedbacks

Audio recordings were transcribed, anonymized, and translated if needed (from Arabic into English for Yemen). The analyst, who was fluent in English and French, used NVivo 11 to code the transcriptions according to the preset themes of effectiveness, efficiency, and participation while remaining open to emerging themes. End-of-workshop evaluations were also analyzed. Tables were used to summarize key facts and figures across the three pilot countries, including recommendations for improvement and lessons learned. These tables allowed iterative comparison across settings to identify common themes and singular perspectives.

## Results

### Effectiveness

By following the step-by-step approach outlined in the toolkit, facilitators enabled participants to produce a work plan at the end of each of the pilot workshops, suggesting that the toolkit was effective in reaching its primary objective. Additionally, results in Yemen suggested that the workshop contributed to raising the importance of SRH, which was perceived to be neglected among decision-makers and health managers and overshadowed by other sectors.

*Previously, it was all about nutrition and other sectors. Reproductive health was totally forgotten. It was a period where reproductive health services were stopped. But we thank them [funders and organizers] for re-activating reproductive health services through their support. —* Participant in Yemen.

In addition to improving comprehensive SRH services in general, the main recurrent themes in work plans were strengthening the healthcare workforce, which was common to all countries (examples included training on comprehensive SRH, building the capacity of midwives, and addressing staff turnover); increasing community mobilization and engagement as well as focusing on adolescent SRH in the Kasai region and Cox’s Bazar; ensuring a sustainable supply chain in the Kasai region and the all three Yemeni Governorates; and expanding maternal and newborn health services in Cox’s Bazar and all three Yemeni Governorates, with an emphasis on quality, coverage, and referral pathways. Priorities specifically highlighted in the Kasai region included renovating health centers, strengthening the health information system, advocating to the government to harmonize the fees for SRH services, and advocating to donors for additional funding. Priorities specifically reported in Cox’s Bazar were expanding the availability of contraceptive services, enhancing demand for and supply of gender-based violence services, and improving the quality of neonatal health services. The capacity development of midwives was a specific concern in Yemen.

### Efficiency

Participants across settings appeared to welcome the methodology and its efficiency in producing results. Participants reported the methodology to be practical and simple and underscored the relevance of using the framework of the WHO Health System Building Blocks. Participants appreciated that a two-day workshop could produce specific and achievable planning priorities within a short time. Most participants across settings found that the workshop duration was adequate; none found it too long. In Sana’a, simultaneous translation added pressure on the available time, and participants felt that the workshop could be longer.

*The methodology was great. It helped us define the priorities we need and how to divide the activities among the six WHO building blocks...It ensured everyone’s participation in planning. –* Participant in Yemen.

*I would like to say that this methodology is more simplified, practical, and participatory in the sense that it was not a colossal and very complex methodology. Everyone had the opportunity to participate in the exchanges. The added value in this workshop is that we started on reliable bases that reflect the real needs in the field … It was the record time that made the biggest impression on me. What impressed me the most was the time that was allotted folks. The whole team came together to identify needs and opportunities in record time. That’s what emergency coordination is all about: responding quickly. You don’t have to take a lot of time while there is an [urgent] need. –* Participants in DR Congo.

Participants found all the successive steps to be useful and complementary. Nonetheless, they proposed concrete changes to enhance the methodology (see Table 2).

**Table 2:** Summary of recommendations to improve the toolkit and its implementation

Themes/Steps	Description
Translation	Where simultaneous translation is required, organizers should plan for at least a 50% increase in workshop duration.
Inclusivity	If key stakeholders – including representatives of often marginalized and underserved populations and communities of concern – are unable to attend the workshop, every effort should be made to include them in the preparation and follow-up processes. This can be done through key informant interviews, focus group discussions, and surveys in advance of the workshop and through follow-up consultations on the work plans developed during the workshop.
Application to different humanitarian contexts	While primarily designed to support the transition from MISP to comprehensive SRH after an acute emergency, this toolkit can also be adapted and used in protracted and complex humanitarian settings to expand the range and enhance the quality of available SRH services, which are often limited to a set of minimal services that may not reach all members of the targeted population.
Data preparation	To ensure that the workshop meets its objective in producing a practical and fact-based work plan, the institution(s) responsible for the organization of the workshop should spend at least 4 to 8 weeks to map the status of the MISP implementation thoroughly. The following information would be useful: who is doing what (which MISP and comprehensive services), where (coverage), when (duration), with which resources (sustainability), and encountering which challenges and opportunities (lessons learned). With careful anticipation, facilitators will have data and information assembled and, if possible, shared with all participants at least a week in advance. This advanced information sharing would allow sufficient time for participants to reflect on the SRH situation before the workshop starts. The more detailed information that can be provided for advance review, the more effective the planning process will be during the brief two-day workshop.
Participant preparation	Participants expressed the need for more information about the workshop objectives, the WHO Health Systems Building Blocks, and what constitutes as MISP versus comprehensive SRH programming. Organizers should send a pre-reading list containing essential information and references to all participants. Participants are encouraged to take the MISP Distance Learning Module in advance of the workshop to learn more about the MISP. Such preparation would allow all the participants to start with a common ground on Day 1 of the workshop.
At the beginning of the workshop	It is essential to have all participants present from the very beginning of the workshop in order not to disturb the participatory process or interrupt the group dynamics.
Steps 2 and 3	Based on the group dynamics, organizers should consider running the reflections on needs and opportunities in small groups rather than individually to maintain participants’ attention. Each group should have a whiteboard to help map and categorize the fruits of their collective work according to the health system building blocks.
Steps 4 and 5	Instead of being divided by geographical areas, participants from one area could be mixed up with participants from other areas to enhance the opportunity to learn from different settings. Facilitators should ask participants how they would like to be grouped – by geography, expertise, interest, or another factor.

### Participation

Participants across settings appeared to highly appreciate the usefulness of the participatory, inclusive, and democratic approach of the workshop, which helped yield a consolidated work plan and a sense of ownership and accountability deriving from the positive and constructive chemistry or “melding of ideas.”

*To me, the methodology is great because it involves participation and discussion of different opinions. It also involves the freedom to say and present whatever you want. It also presents a democratic approach where you can criticize, accept, or reject any point. We hope that we can present what is discussed and planned here into real action. –* Participant in Yemen.

*But another very interesting process was that not everybody talked about the same thing and everybody talked about different points. But there was a melding that took place afterward, where each group got information about what the other was doing and allowed each other’s ideas to feed into the other’s to come to an overall conclusion. –* Participant in DR Congo.

### Relevance of chosen contexts

All the pilot settings were recent or current L3 crises at the time of the workshops. In all three countries, the complexity of the humanitarian situation found and the resulting needs to strengthen the health system after the acute phase of the crisis matched strongly with piloting a planning process that was oriented toward health system strengthening. In Bangladesh, the process coincided with the Joint Response Plan planning process occurring the following year for the Rohingya humanitarian crisis, illustrating how the workshops should build upon the existing efforts of the SRH coordination groups in each context.

The timing between the L3 activation in all three countries and the planning workshop was 8 months in DR Congo, over a year in Bangladesh, and close to 4 years in Yemen, which meant that the planning workshop occurred when recovery efforts were already underway in Kasai and Cox’s Bazar. In the case of Yemen, continuous cycles of insecurity and the resulting shrinkage of the humanitarian space required for effective response hampered the recovery efforts. Nevertheless, the pilots demonstrated the relevance and usefulness of the participatory workshops to catalyze planning for comprehensive SRH services even several months after the acute onset of the crisis, such as in Cox’s Bazar and the Kasai region. Additionally, the Yemen experience spoke for the relevance of the approach when applied to protracted situations.

### Defining principles for collective action in DR Congo

Participants in DR Congo found it essential to define by consensus guiding principles for collaborating on the planning before working on the details of the action plan. The principles are summarized in Table 3 and encompass coordination between actors, non-duplication of efforts, evidence-informed programming, equity in population coverage, and continuous learning through communities of practice.

**Table 3:** Guiding principles for collective action proposed by participants in DR Congo

Guiding principles	Description
Collaboration, participation, complementarity, and coordination between the different actors	To achieve this guiding principle, participants highlighted the importance of: - Continuing coordination meetings at the field level and in Kinshasa, - Designating a focal point or champion for each major activity in the work plan, - Supporting all partners involved in the implementation of the work plan by appointing a project manager working in close collaboration with the SRH coordinator and the SRH working group already in place.
Not reinventing the wheel	- For each activity, take stock of what already exists by mapping existing tools, instruments, and protocols, as well as work plans and projects currently implemented, - Harmonize and adapt the different tools, instruments, and protocols to the specificity of the context and activity in question.
Programming based on scientific evidence	Given limited resources and for efficiency reasons, participants found it essential to: - Implement interventions that have proven successful in similar contexts, - Pilot new interventions but with a robust process of monitoring, evaluation, and even research where feasible.
Equity in population coverage	The channeling of resources must focus on activities in the crisis-affected settings and, in particular, on the most affected, marginalized, and vulnerable populations.
Fostering a community of practice	All activities implemented must be continuously monitored and evaluated in order to help the community of partners to learn, progress, and improve programs and the quality of services.

### Adaptation to logistics constraints in Yemen

In Yemen, due to travel constraints, the lead facilitator was not able to go to Aden to conduct the workshop. Therefore, a creative solution emerged in the form of a four-day training of trainers to enable SRH experts and trainers to deliver the workshop. The training of trainers overlapped with the scheduled two-day planning workshop to offer the trainees the immediate opportunity to observe and co-facilitate. In line with the inclusive approach of the workshop, the facilitator adopted a participatory approach to the training of trainers: participants reflected on and practiced the soft skills required for conducting training, including communication, flexibility, creativity, time management, and leadership. They discussed logistics and potential challenges, such as translation, and co-developed with the main facilitator a practical checklist to assist them with the workshop rollout in Aden. The checklist covered roles and responsibilities among facilitators and the support team, a task division sheet, a list of documents needed for the summary, a list of required materials and logistics, and a material checklist. Four of the participants of the training of trainers facilitated the Aden workshop, which occurred 10 days after the Sana’a workshop. The workshop was reportedly successful. In the end, the unexpected logistic constraints equipped Yemen with additional capacity to organize and conduct future planning workshops to transition from MISP to comprehensive SRH services. Concurrently, the global community acquired a training model and new tools to facilitate a two-day workshop nested within a four-day training of trainers.

## Discussion

In 2017, members of the IAWG Training Partnership Initiative started the crafting of the first toolkit dedicated to supporting the implementation of Objective 6 of the MISP – catalyzing participatory planning to transition from the MISP to comprehensive SRH programming. Results from pilots in DR Congo, Bangladesh, and Yemen converged to suggest that the toolkit was effective in catalyzing the production of consolidated work plans for comprehensive SRH, efficient in its methodology considering the workshop’s duration, and inclusive of key stakeholders and decisionmakers. However, any future implementation should engage community members, including those from often marginalized populations. The pilots yielded several lessons learned, such as enhancing pre-workshop data preparation, equipping all participants with adequate knowledge of the MISP, and defining common principles for collaboration, which allowed for successive improvements of the toolkit. In Yemen, logistics constraints called for a creative solution in the form of training selected participants in Sana’a who, in turn, became facilitators for the workshop in Aden.

### Nature of prioritized activities

Each of the humanitarian situations had its own specificities, gaps, and opportunities in terms of the health system building blocks and response to the SRH needs of the population. Priority activities with crosscutting impacts on SRH services and outcomes were high on the agenda, such as ensuring a sustainable supply chain (notably for the hard-to-reach Kasai region or siege and blockade-affected Yemen), rebuilding and restoring destroyed and looted health facilities in the Kasai region, and enhancing human resources for health [24].

The emphasis on common themes, such as strengthening the overall provision of comprehensive SRH services, capacity development of the health workforce, community mobilization, adolescent SRH, and maternal and newborn health services is somehow unsurprising, albeit critical. Trained, motivated, and retained staff form an essential building block of the health system as they enable access to a wide range of information and services [7]. For instance, in Yemen, new community midwives were trained since 2018 to improve coverage and replace those who retired, left to care for their families, quit their job due to prolonged periods of unpaid salaries, or died, among others [25]. The focus in the work plan to continue supporting the development of such cadres builds of these recent efforts and identified opportunities, and was championed by representatives of the national midwifery association who participated in the workshop.

The MISP objectives focus mostly on the supply side of health services, which must complement activities that generate demand, such as community mobilization and involvement [26]. Although adolescents and young people form a large, if not the largest, cohort across low-income and middle-income countries – including in crisis-affected communities – they often do not have access to adolescent-responsive SRH services that address their specific needs [27]. Basic and comprehensive emergency maternal and newborn care is part of the MISP objectives. These services can be challenging to implement with adequate quality, coverage, and effective referrals that must be sustainable during the recovery and redevelopment phases [28].

Other priorities, such as family planning or gender-based violence, were subsumed under the overall plan to enhance a comprehensive SRH service package or specifically underscored, or both. For instance, the highlight in Cox’s Bazar’s plan to increase awareness on gender-based violence and the quality of related services reflected the high needs in this context [29].

### Implications for policy, practice, and research

The collective work plans for comprehensive SRH that participants developed at the end of the workshops are multipurpose. They could help strengthen the implementation of comprehensive SRH information and services and focus attention on key problem areas. If used to feed into an advocacy and resource-mobilization strategy, they could garner support and funding for programs that feed into the overall reproductive, maternal, newborn, child, and adolescent health program.

*Initial* planning for comprehensive SRH should start at the onset of the acute response, and the participatory process proposed in this toolkit should begin as soon as the MISP clinical services are available and accessible and progress towards reaching Objectives 1 to 5 and other priorities of the MISP are underway. This participatory process could also take place when agencies begin longer-term planning with new funding cycles and in preparation for humanitarian appeal processes. The integration of comprehensive SRH services into these mechanisms could contribute to avoiding service delays and ensuring their sustainability.

Operations research is needed to examine, whenever possible, the implementation of the toolkit in a real-time transition from an acute response toward recovery and health system strengthening. Researching the implementation of the toolkit in protracted situations is equally important. In both cases, the question remains whether and how a work plan with priority activities developed in a participatory manner would translate into the concrete implementation of these priorities and contribute to health system strengthening efforts.

The bigger question will be whether and how each of the settings will implement the prioritized activities in terms of advocacy, identification of sustainable resources, and eventually expanded access to quality services that the community will utilize. The toolkit preempted the possible challenge of seeing the work plans remaining without follow-up and implementation by participating stakeholders. Therefore, it included an important step: a post-workshop follow-up process that participants would conduct to ensure that plans are followed through and challenges addressed—seed funding, even if limited as it was the case in the three countries, could help with the initial operationalization of identified priorities. This review and synthesis of the workshop pilots are limited to the toolkit. However, the IAWG community needs to continue learning about the follow-up to the workshops in DR Congo, Bangladesh, and Yemen. For example, who in the three countries led their operationalization? What was the process undertaken? How useful was the process in advancing the implementation of the work plans? How did it strengthen the health system? What were the challenges, breakthroughs, and recommendations to improve the continuum from planning to implementation?

### Ingredients for success

Several factors may have contributed to the success of this toolkit, including the framing by the WHO Health System Building Blocks, which directly focused participants on interventions that could strengthen the health system. However, it is the participatory nature of the two-pronged design process that likely made the toolkit relevant to local specificities and global needs in terms of developing capacity for comprehensive SRH planning. However, do interventions based on participatory design work in healthcare?

The multiphase participation-centered design of the toolkit was likely a critical component in producing anticipated results with efficiency. Our findings contribute to the current evidence, as illustrated by a synthesis of the effect of community-based participation in various settings [30]. The synthesis showed that collaboration among community partners, facilitators, and organizations led to community-level action that enhanced health and wellbeing, while minimizing health disparities. In the process, it also strengthened the capacity of the community in terms of evaluation skills. Another review found that participatory design could ensure that outputs are appropriate culturally and logistically, generate professional capacity and competence in stakeholder groups, result in productive disagreements followed by useful negotiation, increase the quality of outputs and outcomes over time, increase the sustainability of project goals beyond funded time frames and during gaps in external funding, and create system changes and new, unanticipated projects and activities [31].

The pilots were a product of strong international and national partnerships based on valued and respected collaboration. This multi-level support and investment in the initiative likely contributed to the success in the design and implementation of the workshop toolkit. Moreover, the partners involved in this project had both overlapping and differing objectives and delivery timelines. The funding and programmatic synergies through their partnerships allowed better use of funding with consequently more seed funding for actual program implementation and building buy-in for this effort.

### Limitations

To adhere to different timelines and opportunities, the planning for the pre-workshop preparation and workshop implementation was felt to be limited. The time constraints could have compromised the thorough mapping of the situation in preparation of the workshop, as well as limited the members of priority communities whom the planning process could have engaged—resulting planning priorities could have been different. However, local participating stakeholders came from organizations responsible for direct service and implementation and had a sound knowledge of their settings and awareness of the needs of their community, which could have minimized the risk of producing a work plan of less relevance. Further, in humanitarian contexts, actors must be nimble and responsive to varying limitations and opportunities. The rollout of the workshops was reflective of this reality and still shown to be effective in meeting its objectives.

Future rollouts of the toolkit need to consider the high staff turnover in crisis-affected contexts and factor it into the planning process and strategy for work plan implementation. For example, participants in the workshops in DR Congo and Bangladesh who participated in the planning transitioned out of the response by the time the work plan implementation began. For this reason, it is recommended that there be a clear and consistent sharing of information and knowledge about the planned transition towards comprehensive SRH programming and to ensure work plans move forward and responsibility and accountability are shared.

The facilitators of the workshop were those who conducted structured discussions with the participants in Kinshasa and Sana’a. Therefore, social desirability could have been a potential source of bias in the qualitative inputs. Further, there were no structured discussions in Cox’s Bazaar and Aden. However, results from the qualitative interviews appeared to converge overall with those from the written end-of-workshop evaluation, which took place in all settings.

Finally, it would be naive to believe that thanks to this toolkit, the planning for comprehensive SRH will be straightforward, including the implementation of the planned activities. The relief-to-development continuum is complex and often non-linear, with Yemen and the COVID-19 pandemic providing humbling and eye-opening examples. Technical solutions, including this toolkit, even those embracing participatory principles in design and implementation, will often not work unless major determinants could find roots in the settings [32]. Such determinants encompass the respect for human rights and humanitarian access, a demonstration that the acute phase of the emergency is over, and the presence of a legitimate and functioning national governmental structure [33]. Moving from MISP to comprehensive SRH and striving for health system strengthening are contingent on these aspects as well as funding by local and international donor governments [34]. Foreign policy considerations, rather than only technical ones, determine the investments of donors and their longer-term support to the affected populations.

## Conclusions

Almost 25 years after the creation of the IAWG and conception of the MISP, the IAWG Training Partnership Initiative, in collaboration with the WHO Global Health Cluster, has developed and equipped the global SRH community with the first toolkit to support the implementation of Objective 6 of the MISP, as well as training resources on its use. The collaborative efforts in designing the toolkit with combined bottom-up and global contributions will hopefully render the planning process for comprehensive SRH more systematic and efficient. Consequently, affected communities emerging from acute humanitarian situations or living in protracted settings could have sustained access to quality comprehensive SRH information and services that meet their needs.

## Data Availability

Data is available upon reasonable request from the corresponding author.
